# Economics and Motherhood

**Published:** 1920-04-03

**Authors:** 


### ECONOMICS AND MOTHERHOOD.

An article in the *Woman's Leader* entitled "The Importance of Being Childless" commands attention, because it deals with one of the most serious developments in modern characteristics of the growth of the race—namely, the increasing preponderance of recruits from the least desirable recruiting grounds. The best educated, the most physically fit, and those with a sense of the responsibility of parenthood are the very ones who can best build up a healthy population, and yet are so often compelled to forgo the privilege, or at any rate severely limit it, because of the existing social and

**THE PUBLIC WELL-BEING**—(*continued*)

economic conditions. That is a point which has been noted in several bearings in these columns. The article in the *Women's Leader* is better than its title, which indicates a certain cynicism that the article does not corroborate. It regrets that, compared with the gallant campaigns conducted on behalf of the unmarried mother and child, the increasing troubles and difficulties of the married mother should receive so little attention.

The balance of public sympathy is in danger of swinging over in the wrong direction. "Meanwhile," continues the writer,

"if the production of a jolly little pink citizen, well within the bounds of lawful wedlock, were a crime and an outrage against our national prosperity, it could hardly be more heavily penalised than it is at the present day. There may be a comfortable little self-contained house at a moderate rent built by the fairies at the end of the rainbow; but nowhere else is it to be found. Landladies and boarding-house keepers look with the sourest suspicion upon a perambulator; and the poor young mother who has had to endure all the discomfort and expense of a confinement in 'furnished apartments' will certainly make a secret resolution never to face such an ordeal a second time. Yet the fees of a very ordinary nursing home have already risen to unheard of heights; and very many provincial towns are unprovided with a nursing home of any description—whilst the average general hospital, even if it admits private patients, is reluctant to give up a bed to normal maternity cases. Then of all the profiteers who batten upon our daily needs, the greediest and most merciless are those who surround the new-born baby; doubling and trebling the price of his cradle, his pram, his bath, his soap and powder, and flannels and woollies—even his teddy bear and his fluffy rabbit. Small wonder if he frequently accepts the hint so unwillingly given by his parents, and decides not to get born at all until better times shall come!"

Then follows a practical proposal in the shape of the rearrangement of women's position in the labour market, and a demand at any rate for adequate rates of pay for women, so that they may save as much money as possible before marriage. It is also hinted that women might continue to earn during their married life. Of course, many women help to keep their homes going by charing or other things, though it is doubtful if it is for the benefit of the race. The problem is not so easy with the class of women who—well, who do not go out charing. Certain talented women can make money at home by writing, designing, and other things, but the scope for the average educated woman is not wide, and the larger the family (with the greater need for money) the more has the wife to do in managing her house and her children. But, within limits, there are certainly opportunities nowadays for the young married woman to make money towards the arrival of the "jolly little pink citizen." Many are wise enough to do so, though it is doubtful if their unmarried sisters look with a very cordial eye on these economic competitors. The broad fact, however, remains that if the State, under pressure from various quarters, continues to crush the professional classes under economic burdens, there is little that can prevent their numbers from gradually diminishing. It may not be out of place to mention that Russia had no middle class.

